# Effects of Direct Fluorination on the High-Temperature Oxidation Resistance of AZ31 Magnesium Alloy

**DOI:** 10.3390/ma19010156

**Published:** 2026-01-02

**Authors:** Yu Wang, Jae-Ho Kim, Susumu Yonezawa

**Affiliations:** 1 Department of Materials Science and Engineering, Faculty of Engineering, University of Fukui, 3-9-1 Bunkyo, Fukui 910-8507, Japan; wyd23003@g.u-fukui.ac.jp; 2 Cooperative Research Center, University of Fukui, 3-9-1 Bunkyo, Fukui 910-8507, Japan; yonezawa@matse.u-fukui.ac.jp

**Keywords:** AZ31, magnesium alloy, surface fluorination, oxidation resistance

## Abstract

Fluorination has been proposed as an effective surface modification method for magnesium. The high-temperature oxidation behavior and protective mechanism of fluorinated AZ31 magnesium alloys, especially under prolonged isothermal conditions, have not been systematically investigated. In this study, an efficient and safe surface fluorination method that requires no post-treatment was developed to directly fluorinate the surface of AZ31 machining chips using F_2_ gas. By adjusting the fluorination parameters, including fluorine gas pressure, temperature, and reaction time, the content and uniformity of the surface MgF_2_ layer were effectively improved. High-temperature isothermal oxidation tests demonstrated a remarkable enhancement in oxidation resistance after fluorination; specifically, the weight change of the fluorinated samples decreased from 64.65% for the untreated alloy to 0.68% after oxidation at 450 °C for 12 h. To verify the formation of the MgF_2_ layer and its protective mechanism, all samples were systematically characterized before and after heat treatment using XPS, SEM/EDS, and XRD. The results confirm that direct fluorination with F_2_ is an effective approach for improving the high-temperature stability of AZ31 magnesium alloy.

## 1. Introduction

Magnesium and its alloys, the lightest structural metallic materials used in industry, possess a density of only two-thirds that of aluminum and one-quarter that of steel, together with desirable mechanical properties [1,2,3]. These attributes make them highly attractive for lightweight and energy-efficient applications in the automotive and aerospace sectors [4,5,6]. However, magnesium alloys oxidize rapidly at elevated temperatures and can undergo self-sustaining combustion, posing critical safety concerns [7]. Due to their flammability and poor high-temperature oxidation resistance, regulatory restrictions have long limited their use in safety-sensitive environments [8,9]. For instance, the Federal Aviation Administration (FAA) prohibited magnesium alloys in commercial aircraft cabin structures for decades, easing this restriction only after extensive fire-safety assessments [6,10]. Thus, oxidation instability and combustion risk remain major barriers to the broader adoption of magnesium alloys in high-temperature service.

To expand their application potential, it is essential to understand the oxidation behavior and mechanisms of magnesium alloys at elevated temperatures and identify the root causes of oxide-film failure [11]. Once these factors are clarified, effective strategies for improving their oxidation resistance can be developed, enabling magnesium alloys to maintain stable performance during long-term high-temperature exposure. Metal oxidation typically exhibits a characteristic critical temperature [12]. Below this temperature, a metal can form a relatively compact and adherent oxide film, which stabilizes over time and limits further oxidation. The oxidation kinetics follow a parabolic law: a thin oxide forms initially, after which the growth rate decreases significantly. Above the critical temperature, however, the oxide film cracks, spalls, or loses integrity, leading to linear or power-law oxidation behavior and continuous mass gain [2,13]. In our previous work on pure magnesium powders, the critical temperature was found to be approximately 450 °C. Above this temperature, oxidation proceeded nearly linearly and accelerated with increasing temperature. Macroscopically, large amounts of loose white MgO formed; microscopically, the oxide scale was porous and prone to spallation. This poor oxidation resistance originates from the low Pilling–Bedworth Ratio (PBR = 0.81) of MgO, which prevents the formation of a dense protective film [14]. Improving the high-temperature performance of magnesium therefore requires enhancing the stability and compactness of its surface oxide layer.

AZ-series magnesium alloys, key representatives of the Mg–Al–Zn system, offer excellent castability, good mechanical strength, corrosion resistance, and a mature processing infrastructure, making them the most widely used commercial magnesium alloys [15,16]. Their strengthening derives from Al-based solid solution, β-Mg_17_Al_12_ precipitation strength, and grain refinement [17], while Zn further stabilizes the matrix. Despite their broad industrial use, AZ alloys show inferior high-temperature oxidation resistance compared with pure magnesium [18,19]. This is because aluminum has a lower oxygen affinity and cannot form a protective Al_2_O_3_ film on Mg–Al alloys, while low-melting β-Mg_17_Al_12_ and Mg–Zn intermetallics promote selective oxidation and accelerated magnesium evaporation at high temperatures [20]. Furthermore, oxidation resistance deteriorates with increasing Al content. Studies have shown that when the aluminum content exceeds 10%, magnesium alloys undergo rapid oxidation at temperatures as low as 400 °C [2]. Consequently, the high-temperature oxidation resistance of AZ alloys is inferior to that of pure magnesium and further deteriorates with increasing Al content.

Current research efforts predominantly focus on alloying approaches, in which the addition of rare-earth (RE) or Ca elements promotes the formation of protective oxide films with PBR values greater than 1, thereby effectively suppressing oxidation [5,21,22]. These elements also form stable intermetallic compounds with Al, reducing the detrimental effects of β-Mg_17_Al_12_ on magnesium evaporation. Nevertheless, alloying approaches exhibit inherent limitations: they require modification of the bulk composition and microstructure, involve long processing cycles, may introduce adverse effects on mechanical properties, and significantly increase material cost when RE elements are used [23]. In contrast, surface modification alters only the outermost layer, offering lower cost, higher controllability, and minimal impact on bulk properties. However, the high-temperature stability of most existing surface coatings remains inadequate, and studies focused specifically on surface-engineering-based high-temperature oxidation resistance remain limited [23,24].

Among various surface-modification techniques, surface fluorination involves reacting the substrate with fluorine-containing media to form an in situ MgF_2_ protective layer [25,26,27]. MgF_2_ is a highly stable ionic compound with a high melting point, extremely high decomposition temperature, and exceptional chemical inertness, making it one of the most promising candidates for high-temperature and corrosion-resistant coatings [26]. Among fluorination media, F_2_ gas offers rapid reaction kinetics and enables the formation of high-purity MgF_2_ films. As a gas-phase process, it can uniformly treat samples with complex geometries. Nevertheless, studies on direct F_2_-gas fluorination of magnesium and its alloys are scarce, and experimental data on the high-temperature oxidation resistance and thermal stability of MgF_2_ films formed via this method remain limited.

In our previous work, it demonstrated that fluorination using F_2_ gas can effectively enhance the high-temperature oxidation resistance of pure magnesium. Compared with pure Mg, AZ31 alloy exhibits a higher vapor pressure and is strongly affected by the β-Mg_17_Al_12_ and Mg–Zn phases at elevated temperatures, making it easier to catastrophic oxidation. This places more stringent requirements on the stability of the MgF_2_ protective layer. However, systematic studies on the high-temperature oxidation behavior of fluorinated AZ31 are still lacking, highlighting the need to investigate the protective mechanism of fluorination in such multi-phase magnesium alloys. The present study investigates the high-temperature oxidation behavior of AZ31 machining chips. Samples were fluorinated under various processing conditions using F_2_ gas, followed by high-temperature oxidation testing and microstructural characterization. The influence of F_2_-based fluorination on the high-temperature oxidation resistance of AZ31 was systematically evaluated. This study aims to clarify the influence of fluorination processing parameters on the high-temperature oxidation resistance of AZ31 magnesium alloy and to evaluate the effectiveness of the fluorinated layer in enhancing its high-temperature stability.

## 2. Materials and Methods

### 2.1. Sample Preparation and Fluorination Treatment

AZ31 machining chips (AZ31, Standard testpiece, Kanagawa, Japan) were used as the raw material in this study. The experimental AZ31 samples were not subjected to heat treatment before the experiment. Approximately 350 mg of AZ31 was placed into a nickel reaction vessel (internal dimensions: 24 × 32 × 5 mm^3^) for each fluorination experiment. The samples were dried under high vacuum (0.1 Pa) at room temperature (25 °C) for more than 10 h to remove adsorbed species and environmental impurities. After drying, the vessel was heated and maintained at the desired temperature for fluorination. The fluorination conditions, including the applied F_2_ pressure and reaction time, were adjusted according to the specific experimental requirements. After the reaction, residual fluorine was removed by repeatedly passing the gas through alumina until the fluorine pressure decreased below 10 Torr. The reactor was then purged and evacuated several times using high-purity argon (Concentration: 99.9%; Uno Corp, Fukui, Japan) until a vacuum of 0 was reached. Afterward, argon was introduced to atmospheric pressure, and the samples were removed, weighed, and stored under an argon atmosphere. The detailed parameters of the three fluorination experiments are listed in Table 1.

### 2.2. Isothermal High-Temperature Oxidation Tests

To compare the high-temperature oxidation behavior of untreated and fluorinated AZ31, approximately 40 mg of each sample was placed in a resistance furnace and oxidized at 400 °C and 450 °C. The heating rate was set to 30 °C/min, and the tests were conducted in air for 8 h. After oxidation, the samples were cooled naturally in ambient air. For more precise measurement of mass changes, all the samples (approximately 10 mg) were tested using the Thermogravimetric and differential thermal analysis (TG/DTA; TG/DTA 6300, Seiko Instruments Inc., Tokyo, Japan). The TG/DTA experiments were performed under the same conditions as those in the resistance furnace, with an 8 h isothermal holding time. Data were collected every 0.5 s.

### 2.3. Material Characterization

X-ray diffraction (XRD; XRD-6100, Shimadzu, Tokyo, Japan) was performed to identify phase constituents and examine structural changes before and after oxidation or fluorination. Diffraction patterns were collected in the 2θ range of 10–80°. The obtained spectra were compared with standard PDF database entries and relevant literature reports to determine the phase composition and the evolution of characteristic peaks [23,28,29].

X-ray photoelectron spectroscopy (XPS; JPS-9010, JEOL, Tokyo, Japan) was used to determine the surface elemental composition and chemical states of the samples before and after thermal treatment. All binding energies were calibrated using the adventitious carbon C 1s peak at 284.8 eV. Because the contamination carbon signal is typically weak on metallic Mg-based surfaces, the F 1s peak of MgF_2_ at 685.6 eV which derived from a pre-measured pure MgF_2_ standard and consistent with reported values [30] was additionally used for fine calibration. Based on comparisons with literature data, the binding energies of other elements were subsequently determined. It should be noted that Al_2_O_3_ is converted to α-AlF_3_ during fluorination, with an Al 2p binding energy of approximately 75.0 eV [31,32]. Depth profiling was conducted using an Ar^+^ ion sputtering gun at a sputtering rate of approximately 1.88 nm/s.

Scanning electron microscopy (SEM; JCM-6000Plus, JEOL, Tokyo, Japan) combined with energy-dispersive X-ray spectroscopy (EDS; JCM-6000Plus, JEOL, Tokyo, Japan) was performed to examine surface morphology, the integrity and compactness of oxide/fluoride layers, and the distribution of key elements. EDS provided the spatial distribution of Mg, O, F, and alloying elements such as Al and Zn. These data were used to assess the composition and uniformity of the oxidation and fluorination products. The SEM accelerating voltage was set to 15.0 kV.

## 3. Results and Discussion

### 3.1. Fluorination Treatment Experiment of AZ31 Machining Chips

**Figure 1:**
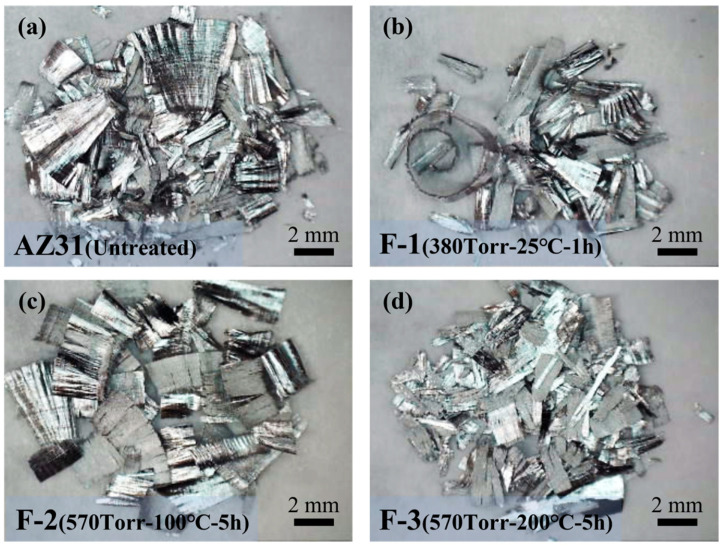
Macroscopic surface morphology of samples after fluorination reaction. [(**a**) Untreated, (**b**) F-1, (**c**) F-2, (**d**) F-3].

**Figure 2:**
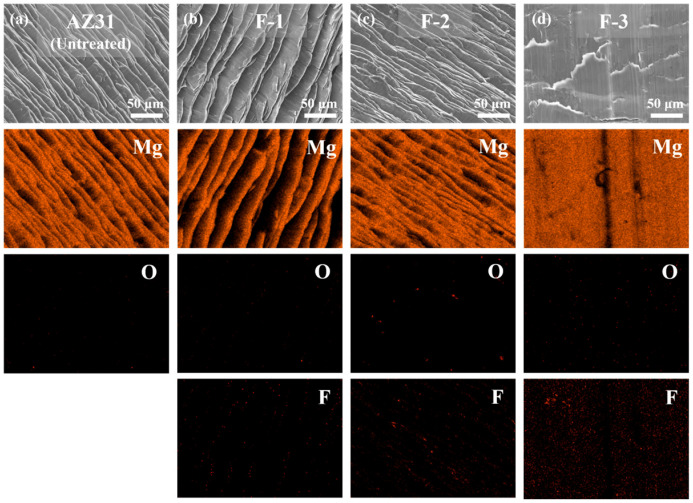
SEM/EDS of samples surface before and after fluorination reaction. [(**a**) Untreated, (**b**) F-1, (**c**) F-2, (**d**) F-3].

Table 2 presents the weight changes of the samples before and after fluorination. The weight remains almost unchanged, indicating that the fluorination treatment does not lead to any measurable weight increase. As shown in the macroscopic observations in Figure 1, all fluorinated samples still exhibit a metallic luster. However, EDS analysis in Figure 2 confirms the presence of fluorine on the surfaces of all fluorinated samples, with sample F-3 displaying the most pronounced fluorine signal. This observation demonstrates that increasing the fluorination temperature, pressure, or reaction time significantly enhances the extent of the reaction between F_2_ and the AZ31 surface.

**Figure 3:**
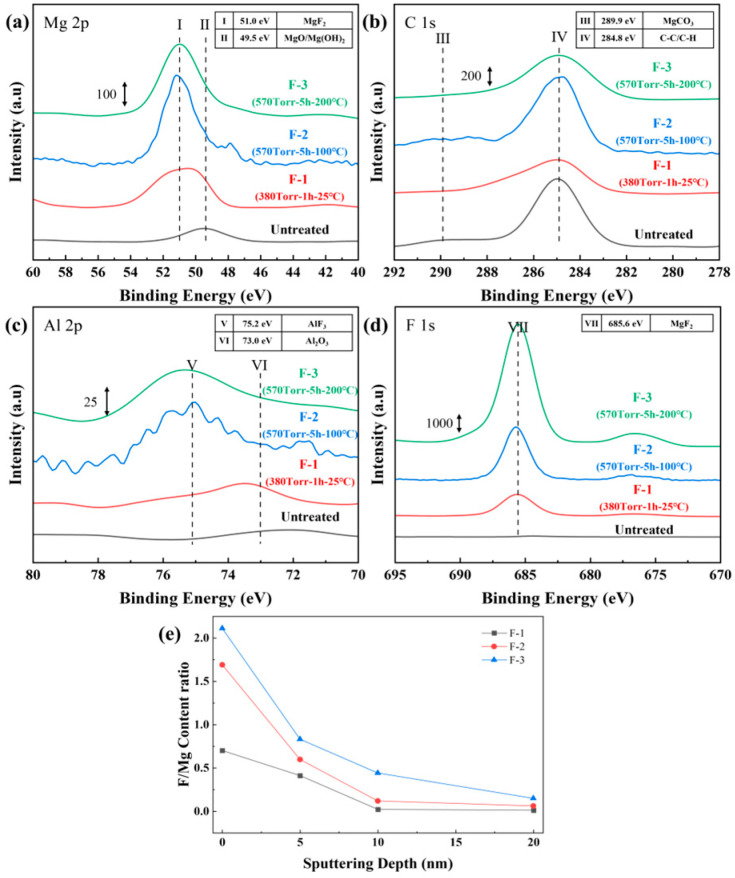
(**a**) Mg 2p, (**b**) C 1s, (**c**) Al 2p and (**d**) F 1s XPS surface analysis of untreated and fluorinated samples. (**e**) The F/Mg ratio at different sputtering depths among the fluorinated samples.

To further identify the fluoride formed on the surface, XPS analysis was performed on all samples. Figure 3 displays the XPS spectra of the main elements, with all spectra calibrated using the carbon C 1s peak at 284.8 eV. In Figure 3c, the Al 2p peak intensity is lower than those of other elements, which is attributed to the low Al content in AZ31 and its weaker oxygen affinity compared with Mg, resulting in a limited amount of surface Al_2_O_3_. After fluorination, distinct binding energy shifts are observed in both the Mg 2p (Figure 3a) and Al 2p (Figure 3c) regions. Meanwhile, the clear F 1s peak is detected in the fluorinated samples (Figure 3d), and its binding energy of 685.6 eV corresponds to MgF_2_. The strong and symmetric F 1s peak further confirms that MgF_2_ is the dominant fluorination product. In contrast, the AlF_3_ peak is not prominent due to the low Al content in AZ31. As summarized in Table 3, the F/Mg atomic ratio increases progressively with optimizing fluorination parameters, reaching 2.11 for sample F-3, indicating substantial MgF_2_ formation on the surface. Figure 3e presents the variation in the F/Mg ratio at different sputtering depths. It can be observed that the F-3 sample maintains a consistently higher F/Mg ratio than F-1 and F-2 within the surface to 20 nm, indicating a higher surface concentration of MgF_2_. However, beyond a sputtering depth of approximately 20 nm, the fluorine signal in all fluorinated samples drops sharply to near-background levels, and MgF_2_ is almost undetectable. This confirms that the fluorination reaction is confined to the uppermost ~20 nm of the surface, characteristic of a surface-limited reaction process.

**Figure 4:**
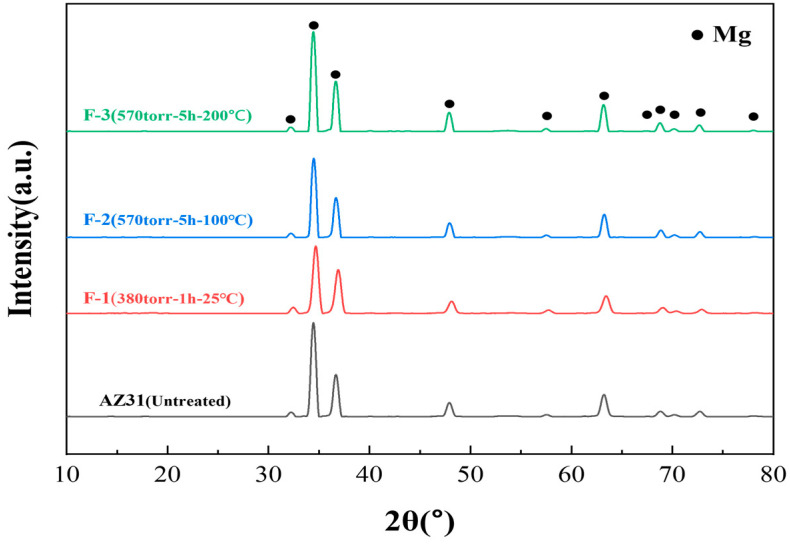
XRD analysis of untreated and fluorinated samples.

The XRD patterns in Figure 4 provide additional evidence that fluorination occurs only at the surface. All samples exhibit peaks corresponding solely to Mg, with no detectable reflections of MgF_2_, suggesting that the fluorinated layer is too thin to be identified by bulk XRD. Similarly, no β-phase (Mg_17_Al_12_) peaks are observed, which is consistent with the low Al content in AZ31 and is similar to previous XRD studies reported in the literature [28].

### 3.2. Isothermal High-Temperature Oxidation of Untreated and Fluorinated AZ31

**Figure 5:**
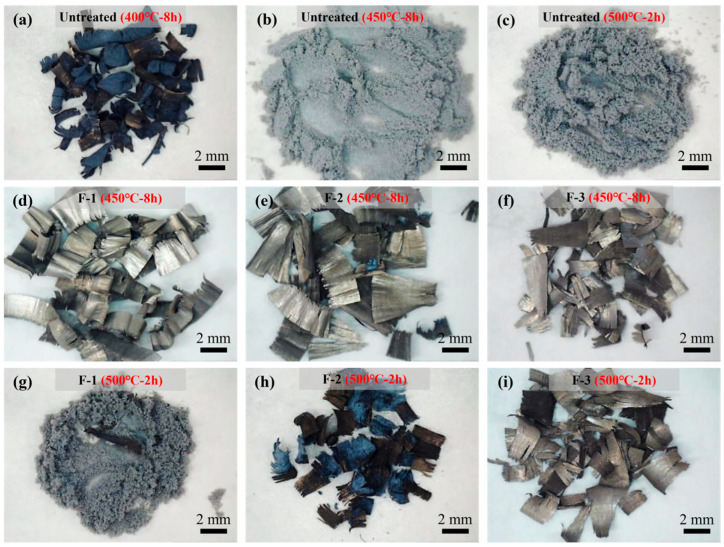
Macroscopic surface morphology of (**a**) UntreatedAZ31 after 400 °C for 8 h, (**b**) UntreatedAZ31 after 450 °C for 8 h, (**c**) UntreatedAZ31 after 500 °C for 2 h, (**d**) F-1 after 450 °C for 8 h, (**e**) F-2 after 450 °C for 8 h, (**f**) F-3 after 450 °C for 8 h, (**g**) F-1 after 500 °C for 2 h, (**h**) F-2 after 500 °C for 2 h, (**i**) F-3 after 500 °C for 2 h.

Figure 5a,b illustrate the macroscopic morphology of the untreated AZ31 alloy after isothermal oxidation at 400 °C and 450 °C for 8 h in a resistance furnace. At 400 °C, the sample had completely lost its metallic luster and developed a black MgO layer, exhibiting oxidation characteristics similar to those of pure magnesium at elevated temperatures. However, the mass gain at this temperature was only 6.14%, indicating that the MgO film still retained a certain oxidation resistance and provided limited protection to the magnesium matrix. In contrast, oxidation at 450 °C resulted in the complete transformation of the sample into a gray powder, with a mass gain of 67.95%, which is close to the theoretical value of 66.67% for the full conversion of Mg to MgO. This demonstrates that the oxide film on AZ31 lost its protective capability entirely at 450 °C, leading to complete oxidation of the magnesium matrix. It was shown that in our previous study on pure magnesium powders [14], even after oxidation at 450 °C and 500 °C for 8 h, pure Mg only formed substantial amounts of white MgO on the surface, with mass gains of approximately 8% and 32%, respectively, without undergoing complete oxidation. In sharp contrast, AZ31 experienced catastrophic oxidation within only 2 h at 450 °C. This obvious difference can be attributed to the presence of the low-melting β-Mg_17_Al_12_ phase and Mg–Zn phases in AZ31, which significantly accelerate Mg evaporation, leading to rapid rupture of the oxide film. Furthermore, large amounts of Mg vapor react vigorously with atmospheric oxygen at high temperatures, releasing substantial heat that further accelerates the oxidation process. In contrast to the untreated samples, the three fluorinated samples (Figure 5c–e) exhibited almost no change in macroscopic morphology after oxidation, with mass gains of only 4.97%, 3.29%, and 3.40%, respectively. These results clearly demonstrate that fluorination markedly enhances the high-temperature oxidation resistance of AZ31. When the temperature was increased to 500 °C, the F-1 sample (Figure 5g) was completely transformed into gray powder, similar to the oxidation behavior of the untreated AZ31 sample shown in Figure 5c. The F-2 sample (Figure 5h) also exhibited pronounced oxidation and noticeable morphological degradation. In contrast, the F-3 sample (Figure 5i) retained its original shape and appearance even under these elevated temperatures. This indicates that F-3 sample maintains excellent oxidation resistance at 500 °C, significantly outperforming the other fluorinated samples.

**Figure 6:**
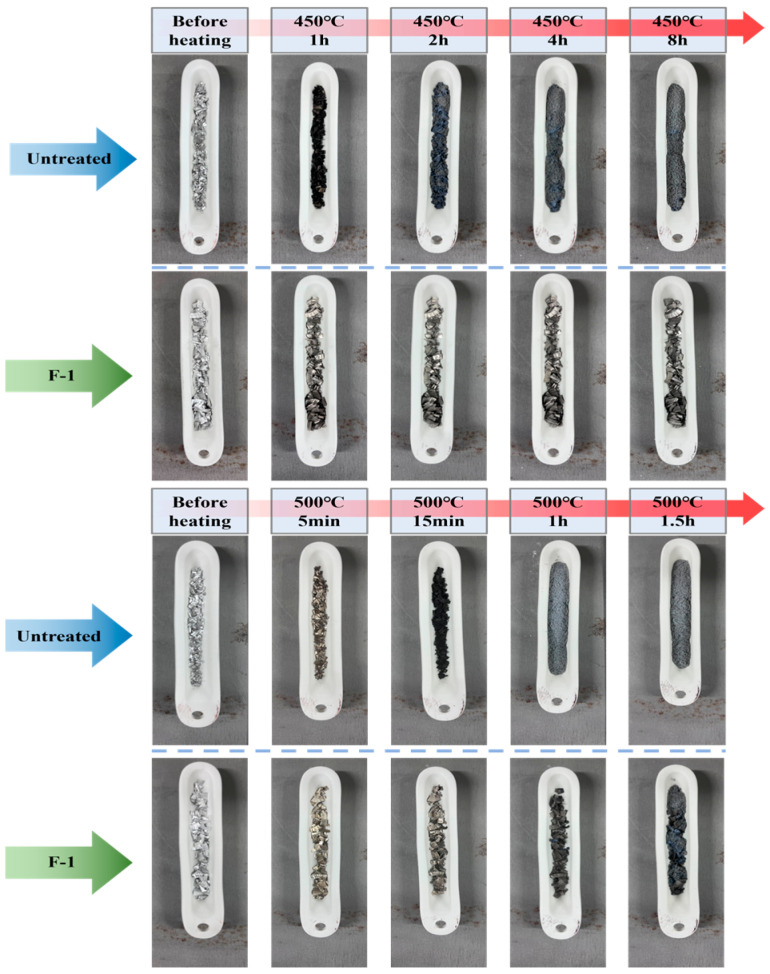
The surface change of untreated sample and the F-1 sample at 450 °C and 500 °C for various time.

Figure 6 compares the oxidation behavior of the untreated sample and the F-1 sample at 450 °C and 500 °C for various time. Under the condition of 450 °C, the untreated AZ31 sample surface turned black after only 1 h of oxidation and transitioned to a gray surface after 2 h. By 4 h, it had almost completely converted into gray MgO powder. In contrast, the F-1 sample showed only a slight decrease in surface gloss after 1 h, with no significant oxidation. Even after 8 h, no noticeable morphological degradation was observed, indicating that the MgF_2_ layer formed during fluorination remains stable and protective during prolonged high-temperature exposure. Under the condition of 500 °C, the untreated AZ31 sample turned completely black within approximately 15 min and was fully oxidized into gray MgO powder after 1 h. This phenomenon is primarily attributed to the extremely high vapor pressure of magnesium at elevated temperatures, which greatly shortens the incubation period of the MgO protective film. As a result, the oxide layer cannot maintain its structural integrity and catastrophic oxidation occurs rapidly. In contrast, the F-1 sample exhibited noticeable black oxidation products only after about 1 h at 500 °C, indicating that the MgF_2_ layer effectively enhances the stability of the oxide film and prolongs the incubation period. However, at such elevated temperatures, the integrity of the protective MgF_2_ layer becomes increasingly critical. To adequately suppress the oxidation reaction, the fluorinated layer must possess high density and uniformity. Once the MgF_2_ film is disrupted—whether by thermal stress or by evaporation—the protective effect is rapidly lost, leading to catastrophic oxidation similar to that observed in the untreated sample.

**Figure 7:**
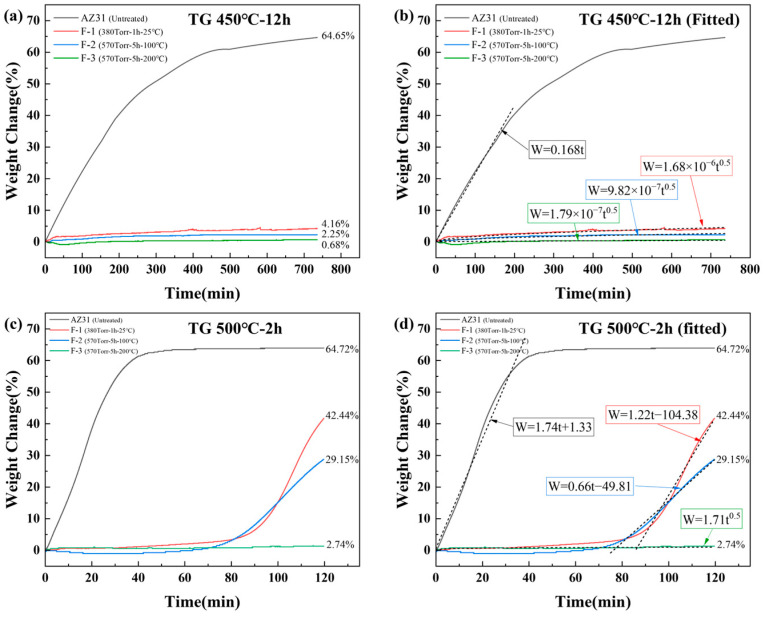
TG of untreated and fluorinated AZ31 samples at (**a**) 450 °C for 12 h and (**c**) 500 °C for 2 h. The Corresponding fitted curves on (**b**) 450 °C for 12 h and (**d**) 500 °C for 2 h.

To obtain more accurate measurements of the weight changes, Figure 7 presents the thermogravimetric (TG) curves and fitted curves of all samples. The heating rate and temperature settings during TG analysis were kept identical to those used in the resistance furnace experiments. As shown in Figure 7a, the oxidation behavior of the untreated sample is consistent with the furnace results, exhibiting a mass gain of 64.65% after 12 h. The untreated AZ31 sample exhibited no incubation period at the initial oxidation stage, indicating that the oxide film provided essentially no protective capability [13]. Due to the high temperature, the initially formed oxide layer was rapidly destroyed, triggering rapid and violent oxidation. This is reflected by the linear and extremely fast mass increase within the first 0–200 min, confirming that the protective film of untreated AZ31 failed almost immediately at 450 °C, leading to catastrophic oxidation. In contrast, the three fluorinated samples exhibited parabolic oxidation kinetics, demonstrating markedly improved high-temperature oxidation resistance. Among them, F-3 showed the best performance, attributable to its higher MgF_2_ surface content, which resulted in a more stable and protective fluorinated layer. The slight differences between the TG-derived mass gains and those obtained from the resistance furnace experiments are likely due to the evaporation of trace amounts of surface moisture and the decomposition of magnesium carbonate and magnesium hydroxide during heating. In the fitted curves shown in Figure 7b, only the data within the initial 0–200 min were analyzed for the untreated sample, as it had completely transformed into MgO powder during the later stages of oxidation. The mass gain of the untreated AZ31 exhibits a clear linear relationship with time, indicating that the oxide film provides essentially no protective effect. In contrast, all three fluorinated samples follow parabolic oxidation kinetics, demonstrating that the MgF_2_ layer offers effective protection at 450 °C. Furthermore, the parabolic rate constant decreases progressively with improved fluorination parameters, confirming the enhanced oxidation resistance resulting from a more uniform and denser MgF_2_ surface layer.

At 500 °C (Figure 7c), the oxidation rate of untreated AZ31 increases by an order of magnitude, and the sample is fully oxidized into MgO powder within approximately 40 min. For the F-1 and F-2 samples, the mass remains nearly unchanged during the initial oxidation stage, but the curves gradually transition to a linear trend at later oxidation stage. This behavior suggests that the MgF_2_ layer is progressively degraded by the high vapor pressure of magnesium at elevated temperatures, leading to a loss of protective capability. Linear fitting of the later-stage curves for F-1 and F-2 (Figure 7d) shows that their oxidation kinetics also follow a linear relationship, similar to the untreated sample, although at a significantly reduced oxidation rate. This indicates that the remaining MgF_2_ still suppresses oxidation to some extent by impeding magnesium ion diffusion. Notably, the F-3 sample retains parabolic oxidation behavior even at 500 °C, with a remarkably low mass gain of only 2.74%. This 500 °C-2 h performance is comparable to that of rare-earth–alloyed magnesium materials (Mg-1.5%Y-0.25%Sn) reported in the literature under similar conditions [33], demonstrating that optimized fluorination treatment can endow AZ31 with high-temperature oxidation resistance on par with rare-earth-strengthened systems.

**Figure 8:**
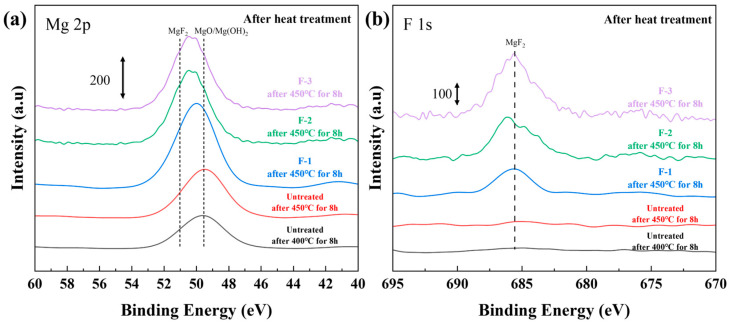
(**a**) Mg 2p and (**b**) F 1s XPS profiles of untreated and fluorinated samples after heat treatment.

Figure 8 shows the XPS results for the samples after heat treatment. Compared with the untreated AZ31, all three fluorinated samples exhibited detectable MgF_2_ signals, and the F 1s peak intensity of F-3 was higher than those of F-1 and F-2 (Figure 8b). When considered together with the TG data shown in Figure 7, this finding indicates that a higher surface MgF_2_ content leads to a more effective protective layer and thus improved high-temperature oxidation resistance. Although the surface MgF_2_ content decreases significantly after high-temperature oxidation, as indicated by the comparison of the data in Figure 3 and Table 3, the TG results in Figure 7 show that the fluorinated samples rapidly form a stable protective layer during the initial oxidation stage, after which their weight remains nearly unchanged. Given the excellent thermal stability of MgF_2_, this behavior suggests that the oxidation resistance is not solely determined by the amount of MgF_2_ remaining on the surface. Instead, the synergistic protection provided by MgF_2_ and MgO at elevated temperatures is likely to play a dominant role. The combined fluorinated–oxidized layer effectively suppresses ion diffusion across the surface and accommodates the internal stresses generated by Mg vapor during oxidation, thereby maintaining long-term protective capability. Table 4 summarizes the elemental compositions of the sample surfaces after the high-temperature treatment. The Al content remained consistently low, confirming that Al possesses a weaker affinity for oxygen than Mg at elevated temperatures and therefore cannot readily diffuse to the surface to form a preferential Al_2_O_3_ protective layer.

**Figure 9:**
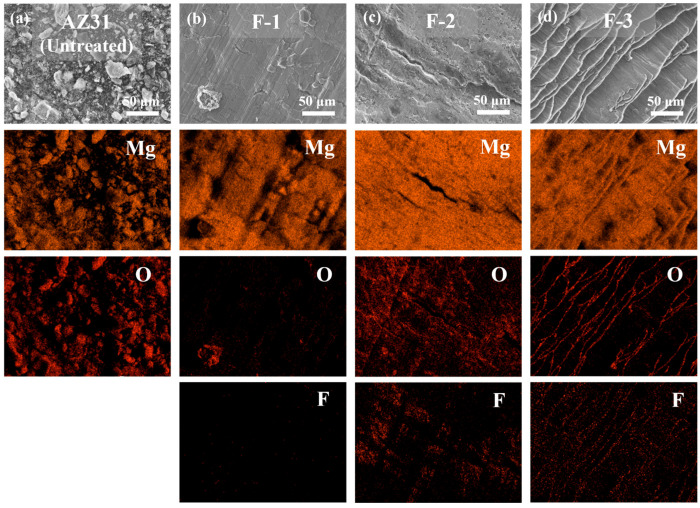
SEM/EDS results of the sample surface after 450 °C for 8 h. [(**a**) Untreated, (**b**) F-1, (**c**) F-2, (**d**) F-3].

In the SEM/EDS analysis shown in Figure 9a, the untreated sample oxidized at 450 °C for 8 h exhibited a fully granular MgO surface morphology, and no detectable Al signal was observed owing to the low Al content in AZ31. In contrast, both the F-2 and F-3 samples showed a substantial and uniformly distributed fluorine signal on the surface. Although the XPS results in Figure 8 indicate that the surface fluorine content decreases after high-temperature oxidation, the uniformly distributed MgF_2_ and MgO in the fluorinated samples act synergistically to impede further oxidation, thereby effectively preventing the progression of the oxidation reaction.

## 4. Conclusions

1. The high-temperature oxidation resistance of the AZ31 alloy is significantly inferior to that of pure Mg, primarily because of the influence of its internal-phase constituents. The low-melting β-Mg_17_Al_12_ and Mg–Zn phases in AZ31 alloys promote accelerated magnesium evaporation at elevated temperatures, resulting in the rapid degradation of the oxide layer. In addition, Al exhibits a weaker affinity for oxygen than Mg and therefore cannot readily diffuse to the surface to form a dense protective Al_2_O_3_ layer. Consequently, the high-temperature oxidation resistance of AZ31 is lower than that of pure Mg. In this study, AZ31 underwent catastrophic oxidation after only 1 h at 450 °C, and prolonged exposure ultimately led to the complete transformation of the alloy into gray-MgO powder. Compared with the materials employed in other studies, machining chips, owing to their larger specific surface area, exhibit more rapid and severe oxidation behavior under high-temperature conditions.
2. In contrast, direct fluorination with F_2_ gas significantly improved the high-temperature oxidation resistance of the AZ31 alloy. By adjusting the fluorination parameters, such as temperature, pressure, and reaction duration, the uniformity and coverage of the surface MgF_2_ layer can be further enhanced. Notably, the F-3 sample exhibited a mass gain of only 0.68% in the TG test at 450 °C for 12 h, compared with 64.6% for the untreated AZ31 sample, demonstrating excellent oxidation resistance. Under the tests conducted at 500 °C for 2 h, the F-3 sample (2.74%) exhibited superior high-temperature oxidation resistance compared with F-1 (42.44%) and F-2 (29.15%).
3. Compared with pure Mg, the phase constituents of AZ31 exerted a more pronounced detrimental effect on the integrity of the protective film at elevated temperatures. Therefore, future work should focus on optimizing the fluorination strategy, such as improving the film density and adjusting the film thickness, to further address the insufficient high-temperature oxidation resistance of AZ31 alloys. Furthermore, fluorination treatment will be carried out on the AZ alloy material of the sheet, and analysis and research will be conducted to better analyze the thickness of the magnesium fluoride layer and calculate the high-temperature oxidation kinetics.

## Figures and Tables

**Table 1:** Sample names and fluorination conditions.

Sample Name	Temperature (°C)	Time (h)	F_2_ Pressure (Torr)
Untreated	-	-	-
F-1	25	1	380
F-2	100	5	570
F-3	200	5	570

**Table 2:** Weight changes before and after fluorination.

Sample Name	Before Fluorination (mg)	After Fluorination (mg)	Weight Changes (%)
F-1	340.88	339.43	−0.43
F-2	338.74	337.33	−0.42
F-3	339.85	339.60	−0.07

**Table 3:** Elemental composition of samples evaluated from XPS results (Figure 3).

Sample Name	Elemental Contents (at %)					F/Mg
	Mg	O	C	Al	F	
Untreated	11.55	30.35	57.00	1.10	-	-
F-1	28.34	25.17	24.41	2.27	19.80	0.70
F-2	21.95	19.92	18.69	2.23	37.21	1.69
F-3	23.76	9.36	14.47	2.23	50.18	2.11

**Table 4:** Elemental composition of samples evaluated from XPS results (Figure 8).

Sample Name	Elemental Contents (at %)				
	Mg	O	C	Al	F
Untreated (400 °C-8 h)	32.36	54.0	10.4	3.11	-
Untreated (450 °C-8 h)	31.55	48.23	16.65	3.57	-
F-1 (450 °C-8 h)	39.87	45.65	8.63	4.59	1.26
F-2 (450 °C-8 h)	30.41	44.32	19.23	3.59	2.45
F-3 (450 °C-8 h)	37.36	36.69	17.99	3.08	4.88

## Data Availability

The original contributions presented in this study are included in this article. Further inquiries should be directed to the corresponding author.
